# Synchronised dual-wavelength mode-locking in waveguide lasers

**DOI:** 10.1038/s41598-018-26152-7

**Published:** 2018-05-18

**Authors:** Wen Qi Zhang, David G. Lancaster, Tanya M. Monro, Shahraam Afshar Vahid

**Affiliations:** Laser Physics and Photonic Devices Laboratories, School of Engineering, The University of South Australia, Mawson Lakes, SA Australia

## Abstract

We present a novel approach to study continuous-wave mode-locking in a waveguide laser in the presence of a gain profile with complex features. We introduce a new simulation approach where we separate the role of gain, nonlinearity, dispersion and saturable absorption elements to provide a better understanding of the interplay between these elements. In particular, we use the simulation to explain synchronised dual-wavelength mode-locking. The results show that despite the existence of dispersion which tends to form separate pulse trains in the laser cavity, the saturable absorber plays a critical role in keeping the different wavelength components synchronised. This work, for the first time, provides insight into existing experimental results. It also demonstrates new methods for studying lasers, especially mode-locking laser, with short laser cavities.

## Introduction

Mode locked waveguide lasers, including fiber lasers, are the most commonly used ultra-short pulse sources due to their high beam quality, high efficiency, and robustness, as well as easy operation. They are used in many application areas such as femtosecond laser machining, spectroscopy, telecommunications as well as frontiers including atomic and molecular sciences^1–5^. The applications drive the development of mode locked waveguide laser towards higher peak-power and narrower pulse-width. In recent years, sub-femtosecond lasers were reported^6–8^. Short pulse widths were achieved with the assistance of strong nonlinear pulse compression and through manipulation of waveguide dispersion.

Theoretical studies on mode-locking mechanism help in understanding and improving the laser performance. Theoretical works have been carried out over past few decades. Most simulations of ultrafast fiber lasers to date have involved Lorentzian gain profiles or their first order approximation for simplicity^9,10^.

However, these models over-simplify the problem for some cases. For instance, the gain profile of Erbium-doped ZBLAN glass can have two peaks centered around 1530 and 1555 *nm* when certain population inversion ratios are applied^11^. The dual-peak gain profile of the erbium-doped glass can lead to dual wavelength mode locking.

Experimental observations of dual-wavelength mode locking have been reported by Zhao^12^ and Huang^13^, with 1530 nm and 1555 *nm* simultaneously mode-locked with two separate pulse trains and switching between the two wavelengths were realised by changing pump power and cavity loss. Similar dual wavelength mode locking behaviors can also be observed under a variety of conditions^14–16^. For most cases, the output pulses for the two wavelengths were temporally separated^12–15^. Temporally synchronised dual-wavelength mode-locking was also reported in a Nd:CNGG crystal laser^16^. It is considered hard to achieve due to the requirement of careful dispersion compensation. Single pulse dual wavelength mode locking has only been observed recently^17^, reporting an erbium-doped ZBLAN glass chip based waveguide laser showing two peaks around 1530 and 1555 *nm* in the output spectrum when the laser is mode-locked while the RF spectrum of the output indicates single pulse operation. The large diameter of the ZBLAN waveguide (∼50 *μ*m) and short length (∼15 mm) indicates no significant contribution of nonlinear effects that could lead to spectral modulation that looks like the measured dual wavelength spectrum, which suggested that the pulse spectrum can only due to the profile of the gain. Furthermore, in that report, the mode-locked laser is operating in the normal dispersion regime without dispersion compensation (ZBLAN has a zero dispersion wavelength around 1.7 *μ*m^18^). A total of 226 *fs*^2^ dispersion is generated at 1550 nm per round trip.

Theoretical work on multi-wavelength mode-locking has been developed by Farnum and Kuts^19^, where a model was built based on coupled wave equations. Each mode-locking frequency was described using one of the equations. Although the model qualitatively explains the dynamics and stability of multi-frequency mode locking. It is not suitable for describing situations such as when the separation between two mode-locking frequency is smaller than the gain bandwidth of each individual mode-locking pulses, as well as mode-locking under a gain profile with complex features such as the gain profile of the Erbium-doped ZBLAN glass mentioned in this paper. The model also has an oversimplified nonlinear absorption term which is not suitable for explaining the complex mode-locking mechanism in Erbium-doped lasers. To the best of our knowledge, there is not yet a model that studied dual-wavelength mode-locking caused by a multi-peaked gain profile.

In this paper, we introduce a novel simulation approach to separate the effects of saturable gain, nonlinearity, group velocity dispersion and saturable loss in each round trip. In this way, we can build a physical understanding of how the interplay of each of the elements influences the development mode-locking. We use this understanding to analyse the dual wavelength mode locking and discover the cause and the dynamics of the synchronization. We believe this is the first time, a numerical approach has been used to investigate the formation of synchronised dual-wavelength mode-locking. Our simulation approach, in general, can be used to understand and develop mode-locked laser.

In our model, we utilized a multi-peak gain model and investigate the effects of nonlinearity, dispersion, and gain on dual-frequency mode-locking. A single master equation which includes all frequencies is used in this study. Using a single master equation does not predetermine the final mode-locking frequency. The final mode-locking frequency establishes naturally through the dynamics of the gain, nonlinearity, dispersion and saturable loss inside of the laser cavity.

The main body of this paper consists of four sections. In Section 2, we lay down the basics of the numerical model. Then we investigate the effects of nonlinearity and dispersion on dual-frequency mode-locking using an artificial dual-peak gain profile in Section 3. In Section 4, we extend the numerical model to include the realistic gain profile of Erbium-doped ZBLAN glass. In the final section of the main body, we introduce a new simulation approach to explore the physics behind the synchronised dual-wavelength mode-locking process.

## The numerical model

**Figure 1:**
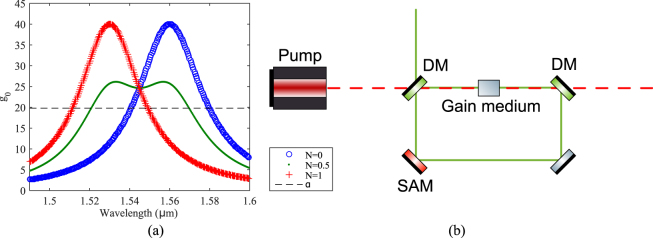
(**a**) Examples of *g*_0_ for *N* = 0, 0.5 and 1, respectively. The horizontal line *α* denotes the cavity loss. Only the part of the gain that is above *α* contributes to the amplification of the laser pulse in the cavity. (**b**) An illustration of the ring cavity used in the simulation. DM: dichroic mirror, SAM: saturable absorber mirror.

The simulation of the mode locking laser is based on the well-known Ginzburg-Landau equation

1 $$\begin{array}{rcl}\frac{\partial u}{\partial z} & = & -i\frac{{\beta }_{2}}{2}\frac{{\partial }^{2}u}{\partial {t}^{2}}+i\gamma {|u|}^{2}u+\frac{g(u)}{2}u,\,g(u)=\frac{{g}_{0}(\omega )}{1+\frac{\int {|u(t)|}^{2}dt}{{E}_{{\bf{s}}{\bf{a}}{\bf{t}}}}},\\ {g}_{0}(\omega ) & = & G(\frac{N}{1+{(\frac{\omega -{\omega }_{1}}{{\rm{\Omega }}})}^{2}}+\frac{1-N}{1+{(\frac{\omega -{\omega }_{2}}{{\rm{\Omega }}})}^{2}})\end{array}$$

where *u*(*t*) is the envelope of the electric field of a laser pulse that contains all frequency components, *z* is the propagation length, *β*_2_ is the group velocity dispersion, *t* is the time in a moving reference frame at speed 1/*β*_1_, *γ* is the nonlinear coefficient, *E*_**sat**_ is the saturation energy of the gain medium, which is set to 50 *pJ*, *g*_0_ is the small signal gain which consists of two peaks, *G* is a constant factor, *ω*_1_ is the frequency of the first gain peak at 1530 *nm* and *ω*_2_ is the frequency of the second gain peak at 1560 *nm*, Ω = 15 THz is the bandwidth of each peak, and *N* is a free parameter between 0 and 1 for adjusting the height of the gain peaks at *ω*_1_ and *ω*_2_ (see Fig. 1a). It worth mentioning that although we use two Lorentzian functions in this example, it is not the only choice and it does not influence the conclusion of this paper.

A fast saturable absorber model Eq. (2) is used for simulating the semiconductor saturable absorber mirror (SAM), where the saturable loss is a function of the instantaneous power of the pulse^20^,

2 $$T(u)=1-{q}_{0}{[1+\frac{{|u(t)|}^{2}}{{P}_{{\bf{sat}}}}]}^{-1},$$

where *T* is the transmission, *q*_0_ is the modulation depth, and *P*_**sat**_ is saturation power.

A linear cavity configuration is used for the simulation, see Fig. 1b. The simulation starts with white noise. The signal passes through the gain medium (15 *mm*) first and then the SAM. Here, we apply an additional 15% (*η*_*i*_ = 0.85) of insertion loss to mimic the non-saturable loss of the SAM and coupling loss before the signal passes through the gain medium again. A 5% out coupling is then applied and the remaining 95% (*η*_*c*_ = 0.95) of the signal is fed back into the loop again. The looping finishes at k’th round trip when the relative change in *u*(*t*) is less than 10^−6^, i.e., $\varepsilon =[\int ||{u}_{k+1}(\omega )|-|{u}_{k}(\omega )||d\omega ]{[\int |{u}_{k}(\omega )|d\omega ]}^{-1} < {10}^{-6}$. In some cases, i.e., when significant nonlinear processes such as self-phase modulation happen during the looping process, strong spectral breathing can occur and the calculation does not converge to the designated value 10^−6^, a maximum number of round trip of 50000 is set. It is important to note that all the results presented in this paper are converged before reaching the maximum round trip number. The SA parameters *q*_0_ and *P*_**sat**_ were set to 8%^17^ and 100 *W*, respectively, corresponding to values in practical scenarios. The 100 *W* saturation power is estimated by assuming 100 *μJ*/*cm*^2^ saturation fluence with a beam spot size of 10 *μ*m in diameter and pulse width of 1 *ps*.

Based on the non-saturated cavity loss, (1 − *q*_0_), 15 *mm* of gain medium length, and insertion losses, *η*_i_, *η*_c_, an absorption coefficient of $\alpha =(1/L)ln{[{\eta }_{{\rm{c}}}{\eta }_{{\rm{i}}}(1-{q}_{0})]}^{-1}=19.8$ *m*^−1^ can be evaluated. The small signal gain *g*_0_ needs to be larger than *α* in order for the signal to be amplified for each round trip. The amplitude of *g*_0_ is determined by the value of *G*, which is determined by the doping concentration of the gain medium. In this work, we pick *G* = 40 which is approximately twice the loss *α* as a starting point. Examples of *g*_0_ are shown in Fig. 1. All data generated from the simulations in the following sections are available.

## Simulation of dual-peak gain laser

Having a gain profile with two distinct peaks, it is intuitive to assume that laser pulses at the two wavelengths propagate at different group velocities when group velocity dispersion exists in the laser cavity. As a result, unless careful dispersion compensation is applied in the cavity design, synchronous mode locking for dual wavelength are not expected to be achieved. However, the experiments of the Erbium ZBLAN chip waveguide laser^17^ demonstrate that synchronisation is maintained, which suggests that this simple explanation does not fully capture the physics behind the experimental observation. In this section, we investigate numerically the impact of group velocity dispersion and nonlinearity on dual-wavelength mode locking.

The symmetrized split-step Fourier method^21^ is used to solve the master equation Eq. (1). The master equation is rewritten in the form

3 $$\frac{\partial u}{\partial z}=(\hat{D}(u)+\hat{N}(u))u,\,\hat{D}(u)=-\,i\frac{{\beta }_{2}}{2}\frac{{\partial }^{2}}{\partial {t}^{2}}+\frac{g(u)}{2},\,\hat{N}(u)=i\gamma {|u|}^{2}.$$

The symmetrized scheme is then applied to give the following relations for iterative propagation

4 $$\begin{array}{rcl}v(z,t) & = & \exp (\frac{h}{2}\hat{D}(u(z,t)))u(z,t),\\ w(z,t) & = & \exp (h\hat{N}(v(z,t)))v(z,t),\,u(z+h,t)\approx \exp (\frac{h}{2}\hat{D}(w(z,t)))w(z,t).\end{array}$$

A time window of 20 ps is used to fit the laser pulse. There are 2^12^ sample points across the time window, which results in a frequency window of 204.8 THz. A step size of 10 *μ*m is used. The initial electric field of pulse *u*(*t*) is populated with white noise, with a noise strength of 10 nW. The laser pulse grows from the white noise until the convergence condition *ε* < 10^−6^ is met. The exact value of the initial noise is not important. Usually, the larger the initial noise, the quicker the pulse starts to deplete the gain.

**Figure 2:**
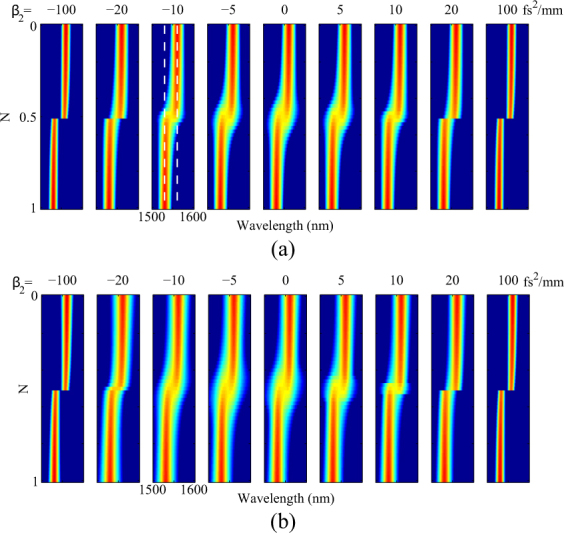
Spectral evolution of mode-locked pulse. (**a**) without nonlinearity. The vertical dotted lines denote the wavelengths of the two gain peaks 1530 *nm* and 1560 *nm*. (**b**) with a nonlinearity of 5 W^-1^ km^−1^

Figure 2 shows the output spectral profile of the mode-locked pulse for different *N* and group velocity dispersion and for *γ* = 0 (a) and *γ* = 5 W^−1^ km^−1^ (b), respectively. Graphs for different dispersion values show similar behaviour for *N* = 0 and *N* = 1. When the dispersion is zero, the peak of the mode-locked pulse is at 1560 *nm* for *N* = 0. As *N* increases from 0 to 0.5, the second peak at 1530 *nm* gradually starts to appear as the gain of the second peak rises above the loss threshold. For values of *N* larger than 0.5, the behavior of the two peaks reverses. The first peak disappears quickly as the gain at 1560 *nm* retreats below the loss. Dual-wavelength mode-locking only appears within a small range around *N* = 0.5. Let us denote this range as Δ*N*. In the figure, dual-wavelength mode-locking is presented for *β*_2_ = −10 to 10 fs^2^/mm around *N* = 0.5 where 1530 and 1560 *nm* peaks co-exist.

When dispersion increases in both negative or positive directions, the spectral width of the output pulse narrows for all *N* values and Δ*N* become smaller until the dual-wavelength mode-locking behaviour ceases to occur. In the case of Fig. 2a, dual-wavelength mode-locking only occurs for absolute dispersion smaller than 20 fs^2^/mm. For large dispersions (larger than ±20 fs^2^/mm in this case), the output pulses only have one peak in their spectrum, either at 1530 or 1560 nm, for all *N*. The mode-locking wavelength switches between the two peaks of the gain when changing *N*.

The presence of nonlinearity extends Δ*N* in the region ${\beta }_{2} < 0$ as the *β*_2_ for maximum Δ*N* move towards negative value in Fig. 2b. For a typical nonlinearity value (such as 5 *W*^−1^*km*^−1^), normal dispersion (${\beta }_{2} > 0$) tends to reduce Δ*N* (comparing to the case without nonlinearity), but in anomalous dispersion regime, Δ*N* increases. The underlying physics that leads to this effect can be explained as follows: The primary nonlinear process that contributes to the gain is modulation instability (MI)^22^. MI provides a maximum gain of 2*γP*_0_ at a frequency shift of $\pm \sqrt{\frac{2\gamma {P}_{0}}{|{\beta }_{2}|}}$. We notice the maximum Δ*N* locates approximately at *β*_2_ = −5fs^2^/mm, which corresponding to a frequency shift of 26 *THz* or roughly 33 *nm* in wavelength. This number aligns well with the separation of the two peaks of the gain. MI only happens in the anomalous dispersion regime. In the normal dispersion regime, four-wave mixing happens instead of MI, which has a frequency shift much larger than the MI and thus does not contribute to the laser gain.

For all the results shown above, we observe only one pulse in the cavity which indicates that synchronous dual wavelength mode locking can exist even when the dispersion is not zero, most significantly at *N* = 0.5. This suggests the existence of another mechanism that cancels out the walk-off between 1530 and 1560 nm peaks. This mechanism is relatively weak dual wavelength mode locking only exists for small dispersion values for either *γ* = 0 or *γ* ≠ 0.

**Figure 3:**
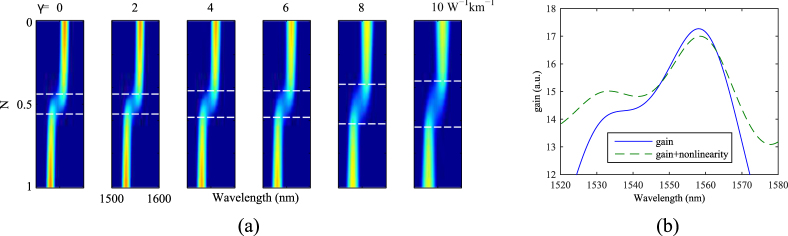
Mode locking under the influence of nonlinearity with no dispersion. (**a**) Mode-locked pulse’s spectral evolution. The white dotted lines indicate the range of Δ*N*. (**b**) The effect of nonlinear reduces the difference between two gain peaks.

Next, we set dispersion to zero and study the effect of nonlinearity alone on dual-wavelength mode locking. Similar to the previous section, we simulate the mode locking for different *N* and nonlinearity values, as shown in Fig. 3a, the figure indicates that nonlinearity has two main effects on the dual wavelength mode locking. Firstly, as expected, the overall bandwidth of the pulse increases as nonlinearity increases. Secondly, the transition range Δ*N* (shown by white dotted lines) increases as nonlinearity increases.

The nonlinearity provides power dependent phase modulation to the laser pulse. It redistributes the pulse energy in the spectral domain as if there is a nonlinear gain similar to the MI gain. The increase of Δ*N* can be explained using this nonlinear gain. When one of the peaks of the gain gets close to the loss threshold as *N* changes, the nonlinearity provides additional gain from the other peak to lift the lower peak above the threshold hence the dual waveguide mode locking happens for a bigger range of Δ*N*. To further explain this, we consider an example, Fig. 3b, in which the gain profiles of material gain alone and combined effective gain (described in Section 5) together with nonlinearity are shown for *N* = 0.4 and *γ* = 4 W^−1^ km^−1^. With material gain alone, the differences in gain between 1530 and 1560 *nm* are large enough that the 1560 *nm* peak dominates and only single wavelength mode locking is possible at *N* = 0.4. However, the presence of nonlinearity reduces that differences (green curve) by taking energy from 1560 *nm* (which acts like loss and therefore the green curve is below the blue curve) to 1530 *nm* (which acts like gain and therefore the green curve is now above the blue curve) to allow dual-wavelength mode-locking.

Similar effects can be observed when dispersion is present. However, the sign of dispersion greatly influences the nonlinear gain. That is why we see different values of Δ*N* in normal and anomalous dispersion regimes as depicted in Fig. 2a.

## Simulation of Erbium doped ZBLAN chip laser

**Figure 4:**
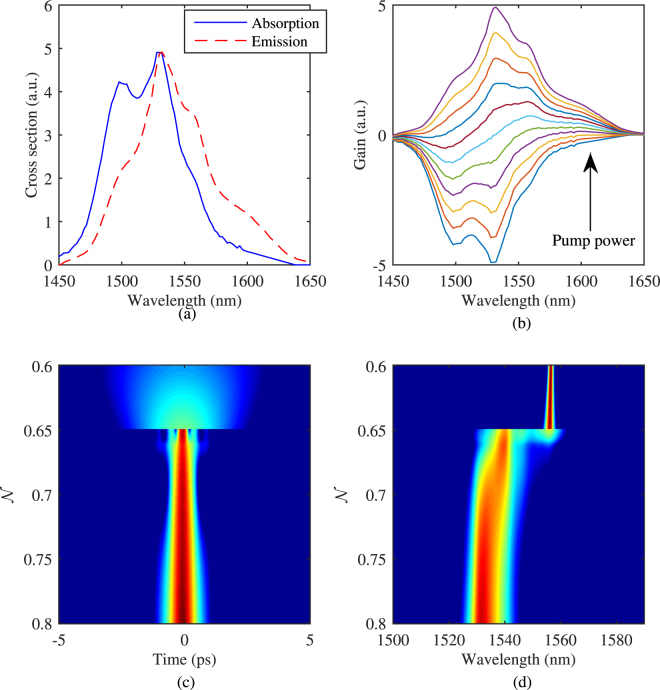
Mode locking in Erbium doped ZBLAN chip waveguides. (**a**) Cross-sections of Erbium doped ZBLAN glass^25^. (**b**) Gain profile as a function of wavelength for different pump power (each line from bottom to top is corresponding to ${\mathscr{N}}$ = 0 to 1 with a step of 0.1). (**c**,**d**) The variation of mode locked output in an erbium doped ZBLAN waveguide laser in temporal and spectral domain for different ${\mathscr{N}}$.

In this section, we investigate synchronised dual-wavelength mode-locking considering a realistic gain profile for Erbium-doped ZBLAN glass, which has a distinct gain and absorption cross-section combination. As shown in Fig. 4a, the emission cross section of the glass has a maximum around 1530 nm, and a small peak around 1560 nm. The absorption cross section of the erbium-doped ZBLAN also has two peaks with the larger peak aligned with the main peak of the emission cross-section. The optical gain coefficient is defined as^11^

5 $${g}_{0}(\lambda )=\rho ({N}_{2}{\sigma }_{e}(\lambda )-{N}_{1}{\sigma }_{a}(\lambda )),$$

where *ρ* is the concentration of the Erbium dopant, *N*_1_ and *N*_2_ are the normalized ion populations in the ground and excited states respectively. *σ*_*e*_ and *σ*_*a*_ are the emission and absorption cross sections respectively. When the laser pump power increases, more ions in the ground state are moved to the excited state, which results in the change of *g*_0_(*λ*). We define ${\mathscr{N}}={N}_{2}/({N}_{1}+{N}_{2})$, and note that changing ${\mathscr{N}}$ is equivalent to change pump power monotonically.

Figure 4b shows the gain coefficient for ${\mathscr{N}}$ ranging from 0 to 1. The negative and positive *g*_0_ means absorption and amplification, respectively. As one can see, for certain ${\mathscr{N}}$ (between 0.6 and 0.7), the long wavelength edge of the absorption cross-section balances out the difference between the 1530 and 1560 *nm* peaks in the emission cross-section and results in gain profiles with relatively equal amount of gain at 1530 and 1560 *nm*. It is expected that dual wavelength mode locking can be observed at these ${\mathscr{N}}$ values the same way as in the modelled two peaked gain profile with N around 0.5.

Simulating the mode-locked laser by using the gain profiles in Fig. 4b, the variation of the mode-locked output (both temporal and spectral profiles) as functions of ${\mathscr{N}}$ are shown in Fig. 4c,d. As ${\mathscr{N}}$ increases from 0 to 1, a sharp transition can be found around ${\mathscr{N}}$ = 0.645 where the pulse width transits from a picosecond regime to a femtosecond regime. This is different from the results of our model for a two-peak gain profile (see Section 3) due to the gain profile of the Erbium-doped ZBLAN glass is not spectrally symmetric. Here the available gain and bandwidth at 1530 and 1560 *nm* are different. At low ${\mathscr{N}}$, the absolute gain value and bandwidth are small, resulting in a narrow-band pulse in the spectral domain and a long pulse in the time domain. Immediately after the transition, large bandwidth pulses with two spectral peaks are obtained. At this point, the output pulse width is minimum. Further increase of ${\mathscr{N}}$ makes the 1530 *nm* peak of the gain become dominant. The bandwidth of the gain in this regime reduces slightly despite the amplitude of the gain still increasing (the bandwidth is defined as the full width at half maximum). The corresponding pulse width enlarges slightly but remains in the femtosecond regime.

**Figure 5:**
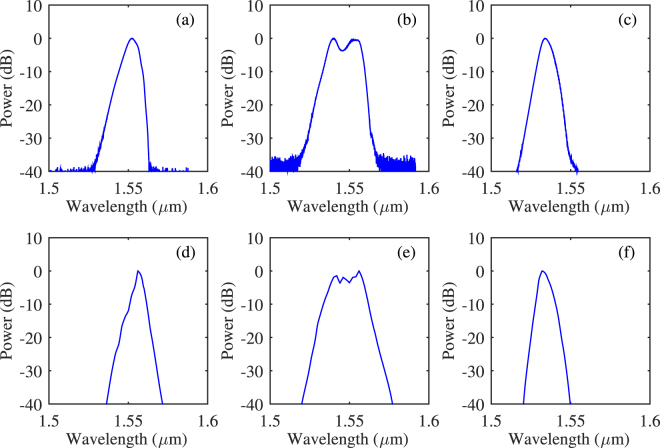
A comparison of experimental observations and numerical simulation. (**a**–**c**) are measured spectra, (**d**–**f**) are calculated spectra.

The simulated results are rather close to the experimental observations. An example of comparison is shown in Fig. 5. The top row of the figure, Fig. 5a,c, are the measured spectra of the laser output at different pump power from low to high. The bottom row Fig. 5d,f are simulated spectra for ${\mathscr{N}}$ equals to 0.6, 0.64 and 0.8. The value of the pump power is not important here since (1) the laser configuration of the laser is linear in reference^17^ whilst it is circular in the simulation, (2) the laser output is also highly sensitive to the alignment of the optics in the cavity (intra-cavity loss). Hence, the actual pump power inside the waveguide and the intra-cavity loss are unknown. However, the behavior of the laser output is the same in general.

**Figure 6:**
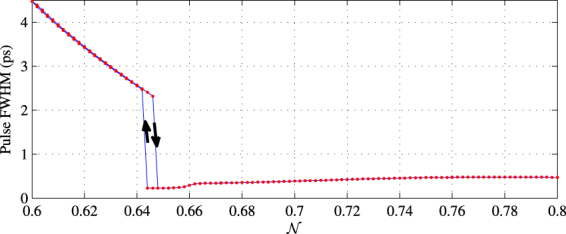
Hysteresis in pulse width with respect to the change of ${\mathscr{N}}$.

Furthermore, unlike the model gain profile Eq. (1), the asymmetrical gain profile of the Erbium-doped ZBLAN glass leads to complex behaviour in the mode locking process. For example, hysteresis is observed in pulse width with respect to ${\mathscr{N}}$. Figure 6 shows the pulse width as a function of ${\mathscr{N}}$. While the laser is mode-locked, changing ${\mathscr{N}}$ from 0.6 to 0.8 and 0.8 to 0.6 result in two different paths for pulse width around the transition region. We think the hysteresis occurs naturally since the loss profile of the saturable absorber depends on the temporal profile of the pre-existing pulse inside the cavity, which in turn affects the available gain of the laser.

The simulation of the chip laser shows qualitative agreement with our ideal model in the previous section. Here, we are interested in the dual wavelength mode locking region. When a dual peak gain profile is presented, synchronous dual wavelength mode locking can be achieved even if the cavity dispersion is not compensated. There must be another mechanism apart from dispersive and nonlinear interactions that bind the two spectral peaks together in time. In the following section, a new simulation approach is proposed for studying this phenomenon.

## A new picture of mode locking

**Figure 7:**
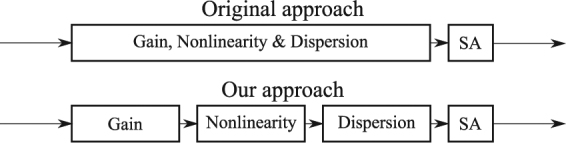
Illustration of our new simulation approach for understanding the mode locking mechanism.

Here we propose a “split-step-like” approach to model the mechanism of synchronously dual wavelength mode locking. In our approach, the pulse propagation through a gain medium with nonlinearity and dispersion is separated into three separate propagation stages; gain-only, nonlinear-only, and dispersion-only, as shown in Fig. 7. Both the new approach and the original approach require the pulse to pass through a saturable absorber after the gain medium, see Fig. 7. In this way, we can study the effect of gain medium and its nonlinearity, and dispersion separately.

Our approach is valid since the length of the waveguide is short and a large number of round trips are required for stable laser output. We have evaluated the loss of accuracy between our approach and the original one and found a negligible difference of only 0.76% in the pulse energy once the mode-locking is established.

Using the proposed approach, we observe the effects of each element of the Ginzburg-Landau equation. For each step in our approach, we define an effective gain and an effective transmission function as

6 $${g}_{{\bf{eff}}}^{X}(\omega )=2\frac{ln\frac{|{\tilde{u}}_{0}(\omega )|}{|{\tilde{u}}_{i}(\omega )|}}{L}\,\,{T}_{{\bf{eff}}}^{X}(t)=\frac{|{u}_{0}(t){|}^{2}}{|{u}_{i}(t){|}^{2}}$$

where the ∼ sign denotes the fields in frequency domain, *u*_*i*_ is the pulse before each element and *u*_*o*_ is the pulse after each element, and *X* can be *G* for gain step, *γ* for nonlinear step, *β* for dispersion step or *S* for saturable absorber. The output pulse *u*_*o*_ of each element is the input pulse *u*_*i*_ of the next element. They loop around indefinitely. Once the laser is mode-locked, the spectra of the adjacent pulses in the pulse train coming out of the cavity are identical. However, each element in the cavity alters the pulse spectrum differently based on the different optical processes involved. Using ${g}_{{\bf{eff}}}^{X}$ we can study how each element changes pulses’ spectral shape and eventually cancels out each other within each round trip. The same thing can be studied in the temporal domain using ${T}_{{\bf{eff}}}^{X}$. Note that ${g}_{{\bf{eff}}}^{X}(\omega )$ and ${T}_{{\bf{eff}}}^{X}(t)$ are implicitly related through the phase of the pulse.

**Figure 8:**
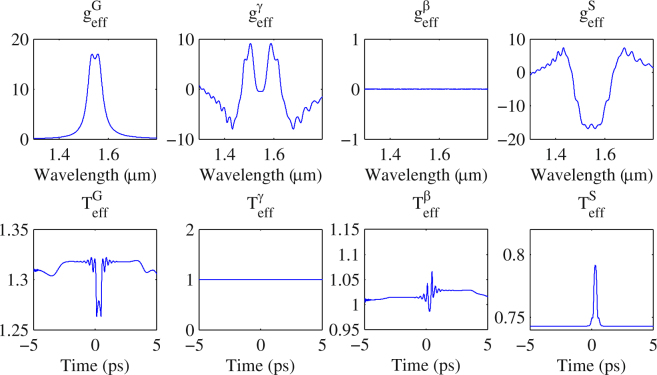
The effective gain and transmission function of each propagation element when CW mode-locking is achieved

When mode locking establishes (*ε* < 10^−6^), the output pulse of the cavity reaches a steady state where in spectrum $\sum {g}_{{\bf{eff}}}^{X}={g}_{{\bf{eff}}}^{G}+{g}_{{\bf{eff}}}^{\gamma }+{g}_{{\bf{eff}}}^{\beta }+{g}_{{\bf{eff}}}^{S}\to 0$. Figure 8 shows an example of the effective gain and transmission functions after each step with *γ* = 5*W*^−1^*km*^−1^, and *β*_2_ = 10 fs^2^/mm.

From Fig. 8, one can see that both gain and saturable absorption alter the spectral and temporal profile of the pulse. However, nonlinearity and dispersion only affect the spectrum and temporal profiles, respectively. Changes of the pulse in the spectral domain cancel out completely in a round trip, once mode-locking is achieved. But in the temporal domain, the pulse shifts slightly with respect to the reference frame of choice of the master equation, which is defined implicitly in the Ginzburg-Landau equation that the reference frame moves along with the group velocity 1/*β*_1_ of the gain medium. When the laser is mode-locked, the $\sum {T}_{{\bf{eff}}}^{X}={T}_{{\bf{eff}}}^{G}+{T}_{{\bf{eff}}}^{\gamma }+{T}_{{\bf{eff}}}^{\beta }+{T}_{{\bf{eff}}}^{S}$ results in a constant shift of the pulse in the time domain as if there is an additional group velocity on top of 1/*β*_1_.

Note from Eq. (2) that the transmission function of the saturable absorber only takes the instantaneous pulse power as the parameter. In other words, the frequency dependence of the pulse does not directly influence the behavior of the SA function. On the other hand, the gain function, *g*(*u*), only takes the pulse spectrum as the parameter and the temporal profile of the pulse does not directly influence the behavior of the gain. Note that the temporal and spectral profiles of the pulse are related through Fourier transform. To reach stable mode locking in the absence of nonlinearity and dispersion, the temporal profile and chirp of the pulse have to be arranged in such a way that the loss generated by the SA compensates those parts of the spectrum that are generated by the gain in a round trip.

In the presence of nonlinearity and dispersion, the pulse spectral and temporal profiles alter, respectively. Nonlinearity transfers energy between different parts of spectrum introducing chirps across the pulse. On the other hand, dispersion stretches and compresses the pulse using chirp as a parameter. Hence, the effects of nonlinearity and dispersion are the redistribution of the pulses’ energy in both the spectral and temporal domain, respectively. In presence of nonlinearity and dispersion, to achieve CW mode locking, the loss generated from the SA combined with any other linear losses, has to compensate changes in the pulse’s temporal (with a shift of the pulse center) profile that has been induced either through dispersion and corresponding frequency changes due to the gain and nonlinearity.

**Figure 9:**
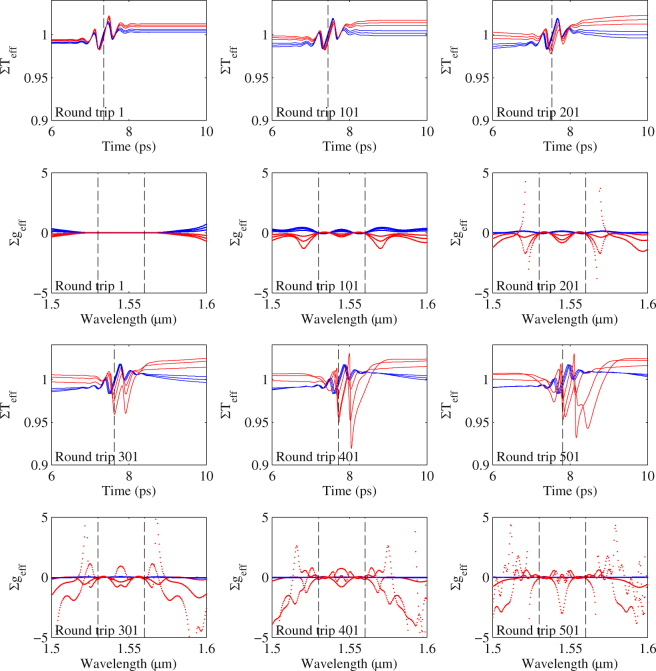
Evolution of the effective gain and transmission with the increase of round-trip number for different dispersion values. Blue curves: with dispersion of 15, 16 and 17 fs^2^/mm, red curves: with dispersion of 19, 20 and 21 fs^2^/mm. The vertical dotted line denotes the center of the pulse.

In the case of dispersion, we notice at a certain dispersion value, i.e. 18 fs^2^/mm in this case, dual wavelength mode-locking disappears suddenly. It is hard to pinpoint the cause of this sudden transition from dual-wavelength to single wavelength mode-locking by looking at the effective gain and transition in the cavity when the mode-locking process is stabilized. The incremental increase of dispersion has unnoticeable changes to $\sum {g}_{{\bf{eff}}}^{X}$ and $\sum {T}_{{\bf{eff}}}^{X}$ in one round trip. However, the small difference in each round trip can be amplified and cascaded through many round trips, which eventually lead to the transition. Figure 9 shows the variations of a pulse ($\sum {g}_{{\bf{eff}}}^{X}$ and $\sum {T}_{{\bf{eff}}}^{X}$) in a round trip at every 100 round trips interval with different dispersion values. The curves are separated into two groups. The blue curves are corresponding to dispersion values smaller than 18 fs^2^/mm (15 to 17 fs^2^/mm), where dual wavelength mode-locking is still possible. The red curves are corresponding to dispersion values larger than 18 fs^2^/mm (19 to 21 fs^2^/mm), where the mode-locking transits to single-wavelength mode-locking. In the $\sum {T}_{{\bf{eff}}}^{X}$ plot, the vertical dotted line indicates the location of the peak of the pulse in the temporal domain.

From Fig. 9, it can be seen that the blue curves with dispersion smaller than 18 fs^2^/mm converge as round-trip number increases. For larger dispersion, the red curves, a loss ($\sum {g}_{{\bf{eff}}}^{X} < 0$) appears in the spectral domain at round trip 101 and then significant modulation appears in $\sum {g}_{{\bf{eff}}}^{X}$, which lead to dramatic changes in pulse’s temporal shape. This observation indicates that the increase of dispersion effectively increases the cavity loss to a point that $\sum {g}_{{\bf{eff}}}^{X}$ can no longer be balanced. The pulse is forced to undergo a significant shape change which results in a different loss profile from the SA to reach a new balance point. However, to explain why the new balance ended up with single wavelength mode-locking, we need to look at the spectrograms of the laser output with different dispersion values.

**Figure 10:**
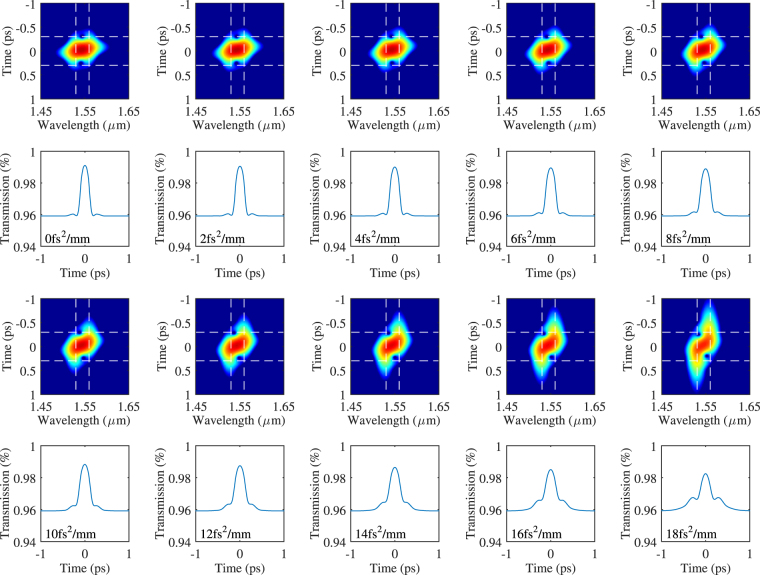
Spectrograms of mode-locking output pulses and the transmission functions of the SA for dispersions from 0 to 18 fs^2^/mm with a step of 2 fs^2^/mm. The vertical dotted lines denote the wavelength 1530 and 1560 *nm*. The horizontal dotted lines are the references for the leading and trailing edges of the pulse.

Spectrograms of the laser output were calculated for dispersion from 0 to 18 fs^2^/mm with a step of 2 fs^2^/mm using a short Gaussian pulse as the gating function. The *T*_0_ width of the Gaussian pulse is 100 fs. The spectrograms are plotted in Fig. 10. The white vertical dash lines mark the locations of the two peaks (1530 *nm* and 1560 *nm*) in the gain spectrum. The horizontal dotted lines are the references for the leading and trailing edges of the pulse.

It can be noticed that as the dispersion increases, a linear chirp was formed. This is seen as a tilt in the spectrograms, where the frequency component of the pulse forms a linear relation with the temporal component. Due to positive *β*_2_, the long (1560 *nm*) and short (1530 *nm*) wavelength sides of the pulse moves into the leading (towards −1 *ps*) and trailing (towards 1 *ps*) edges of the pulse in the temporal domain, respectively. Two horizontal dotted lines are plotted at −0.3 and 0.3 *ps* as references for comparing the extensions of the leading and trailing edges of the pulse. From the spectrograms, one can see the leading and trailing edges belong the spectral components of the two peaks, especially for large dispersion values such as 18 fs^2^/mm. One can consider the chirp formed here is due to the walk-off between the pulses located at 1530 and 1560 *nm*. The larger the dispersion, the further away the two pulses tend to walk off.

**Figure 11:**
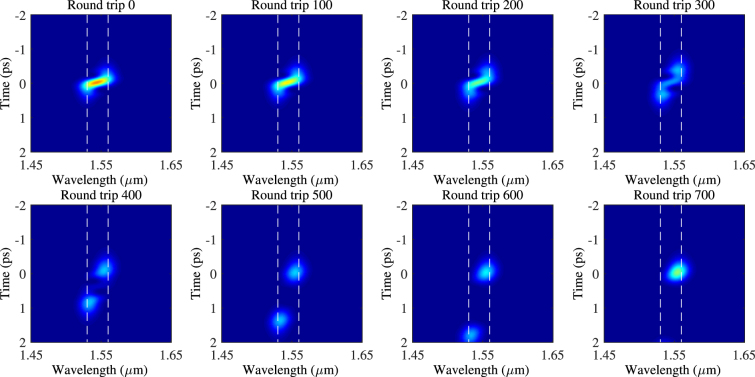
The process of transition from dual-wavelength to single-wavelength mode-locking with *β*_2_ = 20 fs^2^/mm.

As the pulse stretches in the temporal domain, the intensity of the pulse spread over a larger range in time. Thus, the loss generated through SA also increases as SA only take the instantaneous power of the pulse as its parameter. Figure 10 also shows the transmission function of the SA related to the corresponding pulses. The larger the dispersion, the more the pulse spreads and thus the higher loss to the pulse through SA. Furthermore, the leading and trailing edges of the pulse, which correspond to the two peaks of the material gain (1530 and 1560 *nm*), experience higher loss than the center of the pulse. This indicates that although the walk-off between the two mode-locking wavelengths still exists, which is shown as the elongated shape of the pulse in the figure, the ‘walked-off’ portion of the pulses, mainly the leading and trailing edges, are being removed continuously through the loss of SA while the laser gain fills energy back into the pulse resulting in the synchronised dual-wavelength mode-locking output. As the dispersion increases, the SA can no longer remove the ‘walked-off’ portion of the pulse as fast, the leading and trailing edges of the pulse start to gain enough power to compete with the original peak of the pulse and eventually stabilize at a single-peak output. Figure 11 shows the process described above for the case of *β*_2_ = 20 fs^2^/mm.

The key for synchronised dual-wavelength mode-locking lies within the delicate balance between gain, dispersion, and SA. For instance, in order to have synchronised dual-wavelength mode-locking at dispersion values of 20 fs^2^/mm, the modulation depth of the SA needs to be increased to 10% such that a bigger loss difference can be obtained between the peak and edges of the pulse, which enables a fast removal of the energy at the pulse’s edges to keep the two wavelengths synchronised.

## Conclusion

In summary, we numerically investigated the CW mode-locking with a model gain profile consisting of two peaks. By searching through a range of nonlinearity and dispersion values, we found synchronised dual-wavelength mode-locking can be achieved when the heights of the two peaks of the gain are relatively close (30 nm for Erbium doped ZBLAN glass) and the dispersion values are small (|*β*_2_| < 20 fs^2^/mm). Further investigation using a new approach to separate gain, nonlinearity, dispersion and saturable absorber reveals that the SA takes an important role in enabling synchronised dual-wavelength mode locking. The loss profile generated by the SA in the frequency domain has to balance out the spectral gain in the cavity in order to achieve CW mode-locking, whilst the loss profile is determined by the pulse’s temporal shape. In synchronised dual-wavelength mode-locking, the pulse components at the two wavelengths tend to walk-off under the influence of dispersion. However, the loss generated by the SA continuously removes the part of the pulse that has walked off and hence effectively binds the two wavelengths together.

This work, for the first time, explains the synchronised dual-wavelength mode-locking observed in the existing Erbium chip laser experiment^17^. The implication is beyond the scope of this work. As long as the necessary balance conditions in the cavity can be satisfied, stable mode-locking with any pulse shape and spectral profile should be possible. For instance, one can use this principle to engineer the pulse shape of a laser’s output. The link between the temporal shape (dispersion and saturable loss) and spectral profile (gain and nonlinearity) of a pulse is the phase of that pulse. A Gerchberg-Saxton algorithm^23^ may be used to calculate the phase of a given pair of pulse’s profiles in the temporal and spectral domain, and an optical Fourier synthesis technique^24^ can be applied in the laser cavity to help obtain the desired modification to balance conditions for the given pulse shape.
